# Correlation between Preventive Health Behaviors and Psycho-Social Health Based on the Leisure Activities of South Koreans in the COVID-19 Crisis

**DOI:** 10.3390/ijerph17114066

**Published:** 2020-06-07

**Authors:** Young-Jae Kim, Jeong-Hyung Cho

**Affiliations:** Department of Physical Education of Chung-Ang University, Seoul 06974, Korea; yjkim@cau.ac.kr

**Keywords:** COVID-19, leisure, preventative health behavior, mental health, social health

## Abstract

COVID-19 has caused unprecedented damage worldwide, and quarantine and lockdown measures have been undertaken globally. This study focused on the differences in preventive behaviors and psycho-social health of South Koreans, as people continue engaging in leisure activities under self-regulation without a lockdown measure imposed by the government. For the sample, the frame of the “2018 Population and Housing Census” in South Korea was applied, and data from 1770 people were analyzed. The results showed that the groups participating in culture and arts and social activities displayed characteristics with high prevention. Additionally, the groups that continued leisure activities for more than five years and with family showed high preventive behaviors. Meanwhile, participation in leisure activities with friends of the opposite sex lowered preventive behavior. In terms of psycho-social health, all groups were affiliated to the potential stress group and there were no differences in the period and participation time for leisure activities. Furthermore, the group participating in leisure activities with their school and group experienced psychological stability. When lockdown measures are eased, the aforementioned characteristics should be considered to design government policy; they can also be used as a reference for public health in case of a future outbreak of an epidemic.

## 1. Introduction

In December 2019, there was a COVID-19 outbreak [1,2,3] in Wuhan, China, and as of now, 3,267,976 people have been infected worldwide and 233,936 have died [4]. The World Health Organization (WHO) recognized the severity of COVID-19 and declared it a global pandemic, and each nation took strong measures such as quarantine and lockdown to curb the spread of infection [5].

In the case of the United States, the nation with the highest number of cases, quarantine and area lockdown over three consecutive weeks caused loss of employment for 16,800,000 people, which is 11% of the entire workforce; people have also been prohibited from leaving their houses [6]. These measures could reduce the spread of COVID-19, but at the same time, they have resulted in the stigma [7] of democratic rights being curbed and have impeded people’s life balance [8,9]. People’s life balance is closely connected to their work and leisure activities [10], and as people are losing their jobs because of COVID 19 and are prohibited from indulging in various events or leisure activities by the government, the balance between work and life has been destroyed [11]. In the case of South Korea, infected and suspected cases were placed in quarantine. Unlike Italy, China, England, and the United States, the South Korean government recommended that people refrain from social activities, without enforcing a lockdown on the public [12]. The case of South Korea has been cited as the best response to the COVID-19 pandemic [13,14].

With the decreasing number of confirmed cases of COVID-19, the South Korean government is aiming to recover citizens’ regular lives through leisure activities by considering the changes in leisure activities due to COVID-19 [15] and exerting efforts to overcome the disruptions caused by the pandemic [16]. Therefore, research is needed on the ways Koreans are autonomously participating in leisure activities and maintaining the balance between work and life in the current situation.

The previous studies conducted about COVID 19 are mostly related to pathological, virological, and clinical characteristics of the disease [17,18,19]. Although research about the infectiousness of COVID-19 is important, it is also crucial to conduct research that focuses on individual characteristics like the preventive behaviors and psycho-social health of the public.

Human beings have always considered psycho-social measures like preventive behaviors as very important for highly contagious diseases [20]. For instance, a study by Youn and Sook [21] revealed how the level of knowledge about respiratory infection prevention and preventive behaviors are related to the incidence of infection. Furthermore, Tagg and Dierksen [22] and Saiman et al. [23] stated that awareness and knowledge about potential infection and potential infection prevention are related to preventive behaviors against the infection. In particular, Conway et al. [24] emphasized how people’s psychological health functions as an important social factor in coping with a contagious disease like COVID-19. In the global pandemic, people’s involvement in leisure activities can help individuals to recover quickly from emotional scarring caused by the crisis [25] and will also enhance behaviors related to health and influence the quality of life [26].

In response, this study strives to provide basic information about the psychological adjustment mechanism for COVID-19 by analyzing the current status and relationship involving the preventive health behaviors for COVID-19 and psycho-social health based on the social background of South Korean citizens responding to the pandemic as well as the characteristics of their participation in leisure activities.

## 2. Materials and Methods

### 2.1. Participants

The research participants were South Korean citizens, and the frame of the “2018 Population and Housing Census” of the National Statistical Office was applied to select the sample for this study; a total of 3385 subjects were selected through stratified multi-stage cluster sampling. Amongst these selected subjects, 1982 were identified as participating in leisure activities even during COVID-19, and from these the data for 1770 were analyzed, excluding 212 cases with coding misses and inaccurate data. The survey was conducted online by Embrain, the top research company in South Korea. The participants were questioned about whether they participate in leisure activities, and an agreement for participation in the study was procured from those who participated in leisure activities, prior to executing the survey. The study was approved by the Board for Ethical Questions of the University of Chung-Ang (1041078-202003-HR-060-01).

### 2.2. Measurement

#### 2.2.1. COVID-19 Preventive Health Behaviors

For the COVID-19 preventive health behavior scale, the infection preventive behaviors suggested by the Center for Disease Control and Prevention (United States) was used as the basis, and the basic preventive health behavior guidelines provided by the Korean Center for Disease Control and Prevention was used as a reference to apply the measurement instrument by Jong-rim et al. [27] during the Middle East respiratory syndrome (MERS) epidemic.

The abovementioned scale was modified to adapt it to the current COVID-19 situation; for this purpose, advice was provided by one internal medicine physician and one health specialist, and the content validity of the scale was verified. The scale has a total of 11 questions rated on a 5-point Likert scale (5 = “Very likely” and 1 = “Not at all”); a higher score implied a better degree of practice of preventive behaviors. Some of the representative questions include, “refrain from visiting crowded places,” “wear a mask in case of respiratory symptoms like a fever or a cough,” and “ventilate often to maintain clean air inside.” In Jong-rim et al.’s [27] study, the internal consistency reliability of the measurement instrument showed Cronbach’s alpha at 0.770, and the reliability of the instrument used in this study displayed Cronbach’s alpha at 0.838.

#### 2.2.2. Psycho-Social Health 

Data were collected using a questionnaire. For developing the psycho-social health scale, the General Health Questionnaire-60 (GHQ-60) by Burvill and Knuiman [28] was used as the basis. The GHQ-60 includes comprehensive information required to measure the stress level, and has proven to be reliable and valid. For this study, the GHQ-60 was modified to make it suitable to the reality in South Korea. Furthermore, the Psychosocial Well-being Index (PWI) by Se-jin [29] was applied with verification of validation. The PWI consists of 18 questions rated on a 4-point Likert scale. The responses are scored as: 0 = “Not at all,” 1 = “Sometimes,” 2 = “Most of the time,” and 3 = “Always.” The total score ranges from 0 to 54, and higher scores signify an increasingly poorer state of the respondent’s psycho-social health. The scores are classified as: 0−8 for the healthy group, 9−26 for the potential stress group, and 27 and above for the high-risk stress group [30]. In the study by Se-jin [29], the internal consistency reliability showed Cronbach’s alpha at 0.900, and the reliability of the measurement instrument in this study displayed Cronbach’s alpha at 0.867.

#### 2.2.3. Types of Leisure Activities

The types of leisure activities were assessed in this study using the instrument of the Korean Leisure Activities Investigation conducted by the Ministry of Culture, Sports, and Tourism in South Korea once every two years. There are a total of eight types of leisure activities, including ECAA (engagement with culture and art activities, e.g., visiting an exhibition, watching music performances, watching plays, watching movies, etc.), PECA (participating in cultural and art-based activities, e.g., literary events, art activities, playing musical instruments/singing classes, photography, dancing, etc.), WSA (watching sports activities, e.g., live at the sports stadium, or on TV, or indirectly on Digital Multimedia Broadcasting, DMB, etc.), PSA (participation in sports activities, e.g., ball games, racquet sports, winter sports, water sports, swimming, jogging, dance sports, etc.), TOU (tourism, e.g., visit to historical sites, forest baths, camping in the country, overseas trip, visiting natural scenic spots, visiting theme parks, etc.), HEA (hobbies and entertainment activities, e.g., collection activities, crafts, cooking, looking after pets, hiking, surfing online, etc.), RA (relaxation activities, e.g., taking a walk, taking a nice shower, Korean dry sauna, taking a nap, watching television, listening to the radio, listening to music, not doing anything, etc.), and SA (social activities, e.g., voluntary social service, religious activities, clubs, night clubs, visiting family and relatives, talking on the phone, meeting friends, etc.).

### 2.3. Data Analysis

To achieve the objectives of the study, all the analyses of this study applied SPSS version 25.0 followed by coding and data cleaning. A frequency analysis and descriptive analysis were conducted to observe the socio-demographic factors, and Cronbach’s α verification was conducted to verify the reliability of the measurement instruments. Lastly, a one-way analysis of variance (ANOVA) was executed to derive the research results.

## 3. Results

Table 1 displays the characteristics of the participants. Women had higher preventive behaviors (M = 4.245) than men but showed a lower psycho-social health (M = 23.101). The participants in their 40s and 50s comprised the largest proportion of participants (21.6%), but those in their teens (M = 4.205) and 60s and above (M = 4.284) displayed the highest preventive behaviors against COVID-19. Psycho-social health also appeared to be high for those in their teens (M = 20.654) and in their 60s and above (M = 20.655). Among the participants, 44.2% earned more than 4 million Won per month. In addition, 1111 (62.7%) participants were married; married participants showed higher preventive behaviors (M = 4.175) and psycho-social health (M = 22.057) than single participants. In the case of subjective health, 44.6% of the participants considered themselves to be healthy; this group showed high preventive behaviors (M = 4.210) and psycho-social health (M = 18.315). Finally, the score for COVID-19 preventive behaviors was 4.116 on average; regarding the psycho-social health status, 131 (7.4%) people who were rated were included in the healthy group, 1059 (59.8%) in the potential stress group, and 580 (32.8%) in the high-risk stress group.

Table 2 shows the differences in COVID-19 preventive health behaviors according to participation in leisure activities. Among the types of leisure activities, preventive behaviors were highest among those who participated in cultural and art-based activities (M = 4.275, SD = 0.499); it was also shown that preventive behaviors were high (F = 3.694, *p* < 0.001) among the groups that engaged in social activities (M = 4.249, SD = 0.525) and tourism activities (M = 4.223, SD = 0.482). In terms of the period of leisure activities, the group that had participated in these activities for over five years (M = 4.141, SD = 0.503) showed the highest preventive behaviors. For the preventive behaviors based on participation time, the group that participated for 1−2 h (M = 4.149, SD = 0.517) had the highest result (F = 3.549, *p* < 0.014). Regarding the types of groups that participated in leisure activities together, the group that participated with family (M = 4.197, SD = 0.488) showed the highest preventive behaviors (F = 7.735, *p* < 0.001).

Table 3 shows the differences in psycho-social health following the characteristics of participation in leisure activities. Among the different types of leisure activities, social activities (M = 19.421, SD = 9.619) resulted as the highest, and relaxation activities (M = 23.856, SD = 8.238) were the lowest (F = 5.057, *p* < 0.001). There was no significant difference between the period of leisure activities and the participation time for leisure activities. Lastly, among the groups that participated in leisure activities together, those who participated with their school and group (M = 19.609, SD = 9.453) showed the highest result (F = 4.678, *p* < 0.001).

## 4. Discussion

This study investigated the differences in COVID-19 preventive health behaviors and psycho-social health based on the socio-demographic characteristics and the characteristics of participation in leisure activities of the population of South Korea, a country known to have responded well to COVID-19. As a result, preventive actions against COVID-19 were found to be different depending on social characteristics and leisure activity characteristics. It was also found that these characteristics altered the psycho-social health of participants. Specific discussions on this are as follows.

First of all, it was found that COVID-19 preventive behaviors and psycho-social health were higher in women than in men, which is consistent with the results of a study about Australians’ health promotion behaviors based on their lifestyle and gender [31].

The virus affects men and women differently [32]. In the case of the United States, the COVID-19 death rate for men is twice that of women. Meanwhile, in South Korea, the death rate is around 5% higher for men than women [33]. This result can be attributed to women displaying higher preventive behaviors against COVID-19 than men in South Korea. Additionally, women are exposed to more diseases than men. For instance, women are exposed to female-specific diseases such as breast cancer, menopause, and ovarian and cervical disorders [34]. Women are also at a higher risk for many diseases like thyroid and urinary incontinence than men are [34]. Therefore, it is considered that women practice more preventive behaviors for infectious diseases like COVID-19 based on the information and experiences that they have from past diseases. Thus, it is concluded that more education about preventive behaviors should be provided for men to prevent the spread of infectious diseases such as COVID-19.

The COVID-19 preventive behaviors for each age group showed that the age groups in their teens and those in their 60s and above had the highest preventive behaviors. This result suggests that the infection rate is lower in this age group as the preventive behaviors are higher [35]. There are 586 teenage patients infected with COVID-19 in South Korea, which is 5% of the total patients infected, and 22% of those infected with COVID-19 are in their 60s and above. The group in their 60s and above showed a rather high number of infected patients compared to their preventive behaviors, which may be because elderly people with respiratory diseases are more susceptible to COVID-19, so there are more cases among this age group, even if individuals practice exceptional preventive behaviors. Meanwhile, the number of confirmed cases was the highest for the age group in their 20s, with 2940 cases, followed by those in their 40s and 50s [4]. This is consistent with the results of this study regarding the differences in COVID-19 preventive behaviors. In other words, stringent preventive behaviors are essential to prevent the spread of COVID-19, and the results suggest that young adults, in particular, need to be educated regarding preventive behaviors.

With regard to marital status, the results showed that married people had high COVID-19 preventive behaviors. This is consistent with the finding that if one family member is infected with an infectious disease then it could be fatal for the entire family; hence, caution is needed in the actions of all family members [36]. Since COVID-19 is also an infectious disease, those who are married and have a family need to be more careful than those who are single, in order to prevent the disease from affecting their family. In the case of COVID-19, preventive behaviors that were followed were based on the self-rated health condition; those who usually considered themselves as healthy showed high preventive behaviors. In a previous study, it was reiterated through counseling that dietary intake and positive thinking were effective in enhancing a healthy lifestyle [37], and a study by Green and Pope [38] also showed that the mental aspect should be taken into consideration in order to enhance the health condition and prevent health risk behaviors.

There were several differences found in COVID-19 preventive behaviors and psycho-social health depending on the types of leisure activities. The groups with high preventive behaviors against COVID-19 were the groups participating in culture and art, tourism activities, and social activities. This is consistent with the result of the research by Young-sook [39], who analyzed the characteristics of the types of leisure activities.

COVID-19 is an infectious and contagious disease. Accordingly, people who are involved in leisure activities that emphasize the importance of relationships and activities and that involve moving around to other areas are more aware of the risks of transmission of the infection and practice higher preventive behaviors against COVID-19. On the other hand, groups that were involved in relaxation and sports activities showed low preventive behaviors against COVID-19. Recently, the types of leisure activities have been changing due to the COVID-19 situation. People are engaging in leisure activities at home, outdoors, or in their cars without coming into contact with other people [40], and personal leisure activities like taking a walk, going for a run, and jogging have increased instead of those that involve playing sports with others. As a result, people indulging in leisure activities alone at home or outdoors show low prevention against COVID-19, whereas people who engage in leisure activities where people come into contact with others and which involve relationships display high preventive behaviors against COVID-19.

The psycho-social health based on the types of leisure activities showed that all types were potential stress groups. This shows that anxiety is in effect, since everyone carries the possibility of becoming infected by the disease [41]. The stress level appeared to be the highest for those who chose relaxation as their leisure activity, which is because the time that people have to rest at home has increased, causing autonomy to decrease and leading to a new form of stress. Moreover, social activities and gatherings are likely to positively influence people’s psycho-social health. Participation in these activities seems to decrease the self-regulations that make people feel like they are imprisoned, but it also causes a new type of stress.

There were no differences in psycho-social health based on the period and participation time for leisure activities, but there were differences in the preventive behaviors. This partially meets the research findings of Hsieh [42], which showed differences in attitudes depending on the characteristics of participation in leisure activities. Thus, COVID-19 preventive behaviors were high for the group that participated in leisure activities for 1 to 2 h and the group that was engaged in leisure activities for more than five years. This implies that preventive behaviors are practiced more in order to participate in leisure activities.

Lastly, psycho-social health appeared to be higher for the groups that participated in leisure activities with others than people who participated in leisure activities alone. This aligns with the research by Bögels and Emerson [43] which showed that relationships with other people have an influence on individuals’ physical and mental health. In particular, countermeasures like social distancing were reinforced, and people became more careful about maintaining relationships with others while practicing preventive behaviors. Accordingly, people practice preventive behaviors more when they have company, and in this study the group that participated in leisure activities with family showed the highest preventive behaviors. In particular, it is also notable that the groups participating in leisure activities in groups or organizations instead of participating alone scored the highest on measures of psycho-social health. This case displays the cultural communality characteristic [44] in South Korea.

This study focused on the social characteristics of South Koreans and the characteristics of participation in leisure activities amongst various best practices in the unprecedented global crisis due to COVID-19. It investigated COVID-19 preventive behaviors and psycho-social health among the South Korean population and verified the importance of leisure activities for people, even during the pandemic. Nonetheless, the study has the following limitations. First, this is a cross-sectional study and cannot verify causal relationships, so its results should be interpreted cautiously. Second, this study has been conducted while the COVID-19 pandemic is still in progress, so the results could be different for situations before and after the pandemic.

## 5. Conclusions

This study showed the difference in the preventive behaviors and psycho-social health of Koreans during the COVID-19 pandemic, who are participating in leisure activities under self-regulation (recommended by the government) without a lockdown enforced by the government. This shows that while it is important to respond to the infected cases of COVID-19, it is also crucial to take actions as a preventive measure against the disease, stressing the psycho-social phenomenon of the public. In this respect, the group participating in cultural and arts-based activities and the group participating in social activities displayed characteristics with a high prevention against COVID-19, among the leisure activities of Koreans. Additionally, the group that engaged in leisure activities for more than five years and the group participating in leisure activities with their family displayed higher preventive behaviors. Meanwhile, the preventive behavior was low when people participated in leisure activities with friends of the opposite sex. Regarding the characteristics of psycho-social health according to participation in leisure activities, all the groups were part of the potential stress group, and there were no differences in the period of leisure activities and participation time; it was also shown that people experienced psychological stability when participating in leisure activities with their school and group. Therefore, characteristics such as the type of leisure activity, period of participation, participation time, and the presence of company should be considered in order to enhance COVID-19 preventive behaviors in future leisure activities. Once the lockdown is eased and people start participating in leisure activities more, the characteristics studied here can be taken into consideration to design government policy; this information can also be used for future reference for public health in the country and worldwide in the case of a similar epidemic at a later date.

## Figures and Tables

**Table 1:** Characteristics of the participants (*n* = 1770).

Variable	*n*	%	COVID-19 Preventive Behavior: Mean (SD)	Psycho-Social Health: Mean (SD)
**Gender**				
Male	909	51.4	3.995 (0.524) ***	22.216 (8.247) ***
Female	861	48.6	4.245 (0.477) ***	23.101 (9.041) ***
**Age**				
10 s	125	6.4	4.205 (0.514) ***	20.654 (9.009) ***
20 s	320	16.3	4.010 (0.544) ***	22.689 (9.407) ***
30 s	338	17.2	4.020 (0.505) ***	24.041 (8.166) ***
40 s	425	21.6	4.064 (0.510) ***	24.315 (7.672) ***
50 s	426	21.6	4.172 (0.503) ***	21.909 (8.431) ***
60 s and above	334	12.0	4.284 (0.474) ***	20.655 (9.112) ***
**Income**				
Below $ 2650	344	19.7	4.096 (0.534)	24.160 (9.789)
$ 2651~3300	648	36.1	4.115 (0.518)	23.243 (8.350)
Above $ 3301	778	44.2	4.127 (0.508)	21.638 (8.297)
**Marital Status**				
Single	659	37.2	4.018 (0.528) ***	23.329 (8.835) ***
Married	1111	62.8	4.175 (0.501) ***	22.057 (3.669) ***
**Self-Rated Health Condition**				
Good	789	44.6	4.210 (0.508) ***	18.315 (8.665) ***
Fair	813	45.9	4.046 (0.510) ***	25.351 (6.275) ***
Not good	168	9.5	4.021 (0.518) ***	29.898 (8.362) ***
COVID-19 Preventive Behavior	1770	100	4.116	
Psycho-social Health	1770	100		22.646 (8.651)
Healthy group	131	7.4		4.129 (2.573)
Potential stress group	1059	59.8		20.177 (4.718)
High risk stress group	580	32.8		31.337 (4.703)

*** *p* < 0.001.

**Table 2:** Analysis of the differences in COVID-19 preventive behaviors as per participation in leisure activities.

Item	Frequency (*n*)	Average	Standard Deviation	Mean Square	F	Probability	Observed Power	Post Verification
**Leisure Activity Type**				0.978	3.694	0.001	0.978	
ECAA ^1^	286	4.136	0.533					
PECA ^2^	46	4.275	0.499					
WSA ^3^	122	4.069	0.516					
PSA ^4^	129	4.041	0.497					
TOU ^5^	132	4.223	0.482					
HEA ^6^	265	4.139	0.514					
RA ^7^	695	4.073	0.514					
SA ^8^	95	4.249	0.525					
**Period of Leisure Activities**				2.334	8.839	0.001	0.995	a < b, d c < d
Followed by COVID-19 ^a^	48	3.820	0.516					
1−2 years ^b^	183	4.130	0.564					
3−4 years ^c^	216	4.021	0.532					
Over 5 years ^d^	1323	4.141	0.503					
**Participation Time for Leisure Activities (h)**				0.945	3.549	0.014	0.789	α < β
Less than 1 ^α^	263	4.029	0.541					
1−less than 2 ^β^	636	4.149	0.517					
2−less than 3 ^γ^	520	4.131	0.495					
More than 3 ^δ^	351	4.102	0.523					
**Participation in Leisure Activities in Company**				2.038	7.735	0.000	0.998	i, iii > ii
Alone ^i^	727	4.062	0.528					
Family ^ii^	588	4.197	0.488					
Friend of opposite sex ^iii^	108	3.979	0.532					
Friend of same sex ^iv^	237	4.136	0.537					
School & group ^v^	110	4.138	0.472					

Factor description: ^1^ ECAA (engagement with culture and art activities), ^2^ PECA (participating in cultural and art-based activities), ^3^ WSA (watching sports activities), ^4^ PSA (participation in sports activities), ^5^ TOU (tourism), ^6^ HEA (hobbies and entertainment activities), ^7^ RA (relaxation activities), ^8^ SA (social activities). ^a^ Followed by COVID-19, ^b^ 1−2 years, ^c^ 3−4 years, ^d^ Over 5 years. ^α^ Less than 1 h, ^β^ 1−less than 2 h, ^γ^ 2−less than 3 h, ^δ^ More than 3 h. ^i^ Alone, ^ii^ Family, ^iii^ Friend of opposite sex, ^iv^ Friend of same sex, ^v^ School & group.

**Table 3:** Differences in psycho-social health based on participation in leisure activities.

Item	Frequency (*n*)	Average	Standard Deviation	Mean Square	F	Probability	Observed Power	Post Verification
**Leisure Activity Type**				405.014	5.507	0.001	0.999	3, 7 < 8
ECAA ^1^	286	22.185	8.316					
PECA ^2^	46	20.673	9.802					
WSA ^3^	122	23.852	8.492					
PSA ^4^	129	21.558	7.983					
TOU ^5^	132	22.015	9.061					
HEA ^6^	265	21.762	9.148					
RA ^7^	695	23.856	8.238					
SA ^8^	95	19.421	9.619					
**Period of Leisure Activities**				94.667	1.265	0.285	0.341	
Followed by COVID-19 ^a^	48	24.916	6.377					
1−2 years ^b^	183	22.251	8.107					
3−4 years ^c^	216	22.453	7.814					
Over 5 years ^d^	1323	22.650	8.917					
**Participation Time for Leisure Activities (h)**				130.232	1.742	0.156	0.458	
Less than 1 ^α^	263	23.178	8.033					
1−less than 2 ^β^	636	22.772	8.417					
2−less than 3 ^γ^	520	21.953	8.101					
More than 3 ^δ^	351	23.048	10.149					
**Participation in Leisure Activities in Company**				347.295	4.678	0.001	0.951	i, ii, iv < v
Alone ^i^	727	23.295	8.846					
Family ^ii^	588	22.375	8.014					
Friend of opposite sex ^iii^	108	22.324	7.822					
Friend of same sex ^iv^	237	22.886	9.253					
School & group ^v^	110	19.609	9.453					

Factor description: ^1^ ECAA (engagement with culture and art activities), ^2^ PECA (participating in cultural and art-based activities), ^3^ WSA (watching sports activities), ^4^ PSA (participation in sports activities), ^5^ TOU (tourism), ^6^ HEA (hobbies and entertainment activities), ^7^ RA (relaxation activities), ^8^ SA (social activities). ^a^ Followed by COVID-19, ^b^ 1−2 years, ^c^ 3−4 years, ^d^ Over 5 years. ^α^ Less than 1 h, ^β^ 1−less than 2 h, ^γ^ 2−less than 3 h, ^δ^ More than 3 h. ^i^ Alone, ^ii^ Family, ^iii^ Friend of opposite sex, ^iv^ Friend of same sex, ^v^ School & group.

## References

[1] Oyelola A.A., Adeshina I.A., Ezra G. (2020). Early Transmission Dynamics of Novel Coronavirus (COVID-19) in Nigeria. Int. J. Environ. Res. Public Health.

[2] Xiaoru X., Liman H., Jun L., Hong Z. (2020). Generational Differences in Perceptions of Food Health/Risk and Attitudes toward Organic Food and Game Meat: The Case of the COVID-19 Crisis in China. Int. J. Environ. Res. Public Health.

[3] Huang C., Wang Y., Li X., Ren L., Zhao J., Hu Y., Cheng Z. (2020). Clinical features of patients infected with 2019 novel coronavirus in Wuhan, China. Lancet.

[4] CoronaBoard. COVID-19 Dashboard. Last update: 5/1/2020, 10:00:21 AM. https://coronaboard.kr/en/.

[5] Adalja A.A., Toner E., Inglesby T.V. (2020). Priorities for the US health community responding to COVID-19. JAMA.

[6] Anneken T., Annalyn K. Another 6.6 million Americans filed for unemployment benefits last week. CNN Business. 9 April 2020. https://edition.cnn.com/2020/04/09/economy/unemployment-benefits-coronavirus/index.html.

[7] Spengler. Covid-19 is ‘an affront to democracy’. Asia Times. 24 April 2020. https://asiatimes.com/2020/04/covid-19-is-an-affront-to-democracy/.

[8] Holshue M.L., DeBolt C., Lindquist S., Lofy K.H., Wiesman J., Bruce H., Diaz G. (2020). First case of 2019 novel coronavirus in the United States. N. Engl. J. Med..

[9] Khurshid Z., Asiri F.Y.I., Al Wadaani H. (2020). Human saliva: Non-invasive fluid for detecting Novel Coronavirus (2019-nCoV). Int. J. Environ. Res. Public Health.

[10] Kang S.W., Kim Y.J. (2019). A Study on the Keyword Analysis of Warabal (WLB) and Work-life Balance through YouTube. Korean J. Leis. Recreat. Park.

[11] Maurie Backman Is COVID-19 Destroying Work-Life Balance? The Motley Fool. 25 April 2020. https://www.fool.com/careers/2020/04/25/is-covid-19-destroying-work-life-balance.aspx.

[12] Sean Fleming South Korea’s Foreign Minister explains how the country contained COVID-19. World Economic Forum. 30 April 2020. https://www.weforum.org/agenda/2020/03/south-korea-covid-19-containment-testing/.

[13] Michae. Combating COVID-19: Lessons from South Korea. Brookings. 13 March 2020. https://www.brookings.edu/blog/techtank/2020/04/13/combating-covid-19-lessons-from-south-korea/#cancel.

[14] John P. South Korea’s coronavirus response is the opposite of China and Italy—And it’s working. 13 March 2020. This week in Asia. https://www.scmp.com/week-asia/health-environment/article/3075164/south-koreas-coronavirus-response-opposite-china-and.

[15] Lee S.I. Leisure life changed by COVID. Ministry of Health and Welfare, Tasari Press Group. 24 April 2020. https://blog.naver.com/mohw2016/221926058807.

[16] Nam Y.W. Try to overcome COVID-19 positively through leisure activities. Korea Daily News. 31 March 2020. http://www.hyundaiilbo.com/news/articleView.html?idxno=466013.

[17] Zhang Y., Ma Z.F. (2020). Impact of the COVID-19 Pandemic on Mental Health and Quality of Life among Local Residents in Liaoning Province, China: A Cross-Sectional Study. Int. J. Environ. Res. Public Health.

[18] Fang L., Karakiulakis G., Roth M. (2020). Are patients with hypertension and diabetes mellitus at increased risk for COVID-19 infection?. Lancet Respir. Med..

[19] Anderson R.M., Heesterbeek H., Klinkenberg D., Hollingsworth T.D. (2020). How will country-based mitigation measures influence the course of the COVID-19 epidemic?. Lancet.

[20] Ewart C.K. (1991). Social action theory for a public health psychology. Am. Psychol..

[21] Choi J.Y., Park K. (1999). Sook. A Study on the Prevention of Nosocomial Respiratory Infection in Critical Care Nurses. J. Korean Acad. Fundam. Nurs..

[22] Tagg J.R., Dierksen K.P. (2003). Bacterial replacement therapy: Adapting ‘germ warfare’to infection prevention. Trends Biotechnol..

[23] Saiman L., Siegel J.D., LiPuma J.J., Brown R.F., Bryson E.A., Chambers M.J., Downer V.S., Fliege J., Hazle L.A., Jain M. (2014). Infection prevention and control guideline for cystic fibrosis: 2013 update. Infect. Control. Hosp. Epidemiol..

[24] Conway III L.G., Woodard S.R., Zubrod A. (2020). Social Psychological Measurements of COVID-19: Coronavirus Perceived Threat, Government Response, Impacts, and Experiences Questionnaires. Psy. Ar. Xiv.

[25] Paluska S.A., Schwenk T.L. (2000). Physical activity and mental health. Sports Med..

[26] Cho J.H., Kim Y.J. (2016). The Impact of Physical Activity on Quality of Life and Subjective Health. Asian J. Phys. Educ. Sport Sci. (AJPESS).

[27] Choi J.R., Go I.S., Lim Y.E. (2016). Factors Influencing Nursing Students’ Performance of Infection Control. J. Korean Acad. Fundam. Nurs..

[28] Burvill P.W., Knuiman M.W. (1983). Which version of the General Health Questionnaire should be used in community studies?. Aust. N. Z. J. Psychiatry.

[29] Jang S.J. (2000). Standardization of the collection and measurement of health statistics. Korean Soc. Prev. Med..

[30] Kim J.H. (1999). The Reliability and Validity Test of Psychosocial Well-being Index(PWI). J. Korean Acad. Nurs..

[31] Kendig H., Browning C.J., Thomas S.A., Wells Y. (2014). Health, lifestyle, and gender influences on aging well: An Australian longitudinal analysis to guide health promotion. Front. Public Health.

[32] BBCNEWS (2020). Corona19: Why are the effects of the virus on men and women different? BBCNEWSKOREA. https://www.bbc.com/future/article/20200409-why-covid-19-is-different-for-men-and-women.

[33] Korea Centers for Disease Control and Prevention Patient status of COVID-19. 27 April 2020. KCDC.

[34] Dunne E.F., Unger E.R., Sternberg M., McQuillan G., Swan D.C., Patel S.S., Markowitz L.E. (2007). Prevalence of HPV infection among females in the United States. JAMA.

[35] Li Z., Folmer H., Xue J. (2016). Perception of air pollution in the Jinchuan mining area, China: A structural equation modeling approach. Int. J. Environ. Res. Public Health.

[36] Kannan T.R., Hardy R.D., Coalson J.J., Cavuoti D.C., Siegel J.D., Cagle M., Baseman J.B. (2012). Fatal outcomes in family transmission of Mycoplasma pneumoniae. Clin. Infect. Dis..

[37] Lin J.S., O’Connor E., Evans C.V., Senger C.A., Rowland M.G., Groom H.C. (2014). Behavioral counseling to promote a healthy lifestyle in persons with cardiovascular risk factors: A systematic review for the US Preventive Services Task Force. Ann. Intern. Med..

[38] Green C.A., Pope C.R. (2000). Depressive symptoms, health promotion, and health risk behaviors. Am. J. Health Promot..

[39] Song Y.S. (2013). Leisure Satisfaction Differences According to Type of Life Style and Leisure Activity Features. J. Korea Entertain. Ind. Assoc..

[40] Kim J.H., Kim K.Y. I enjoy it at home, in my car...people looking for leisure without contact. 06 March 2020. DongA News. http://www.donga.com/news/article/all/20200306/100043794/.

[41] Coughlin S.S. (2012). Anxiety and depression: Linkages with viral diseases. Public Health Rev..

[42] Hsieh C.M. (1998). Leisure Attitudes, Motivation, Participation, and Satisfaction: Test of a Model of Leisure Behavior.

[43] Bögels S.M., Emerson L.M. (2019). The mindful family: A systemic approach to mindfulness, relational functioning, and somatic and mental health. Curr. Opin. Psychol..

[44] Choi M.S. (2005). Identity of Korean culture, driving force behind Korean Wave. Humanit. Contents.

